# Core-Proteomics-Based Annotation of Antigenic Targets and Reverse-Vaccinology-Assisted Design of Ensemble Immunogen against the Emerging Nosocomial Infection-Causing Bacterium *Elizabethkingia meningoseptica*

**DOI:** 10.3390/ijerph19010194

**Published:** 2021-12-24

**Authors:** Muhammad Idrees, Muhammad Yasir Noorani, Kalim Ullah Altaf, Eid A. Alatawi, Faris F. Aba Alkhayl, Khaled S. Allemailem, Ahmad Almatroudi, Murad Ali Khan, Muhammad Hamayun, Taimoor Khan, Syed Shujait Ali, Abbas Khan, Dong-Qing Wei

**Affiliations:** 1 Center for Biotechnology and Microbiology, University of Swat, Swat 19200, Khyber Pakhtunkhwa, Pakistan; idrees017@gmail.com (M.I.); shujaitswati@uswat.edu.pk (S.S.A.); 2 Nishtar Medical College, Multan 59300, Punjab, Pakistan; Tamimrai18@gmail.com; 3 Kyrgyz State Medical Academy, Bishkek 720000, Kyrgyzstan; habibrai36@gmail.com; 4 Department of Medical Laboratory Technology, Faculty of Applied Medical Sciences, University of Tabuk, Tabuk 71491, Saudi Arabia; eid.alatawi@ut.edu.sa; 5 Department of Medical Laboratories, College of Applied Medical Sciences, Qassim University, Buraydah 51452, Saudi Arabia; Ffabaalkhiel@qu.edu.sa (F.F.A.A.); K.allemailem@qu.edu.sa (K.S.A.); 6 Department of Pharmaceutical Chemistry and Pharmacognosy, College of Dentistry and Pharmacy, Buraydah Colleges, Buraydah 51418, Saudi Arabia; 7 Department of Chemistry, Kohat University of Sciences and Technology, Kohat 26000, Khyber Pakhtunkhwa, Pakistan; drmalikhan@yahoo.com; 8 Department of Botany, Abdul Wali Khan University, Mardan 23200, Khyber Pakhtunkhwa, Pakistan; hamayun@awkum.edu.pk; 9 Department of Bioinformatics and Biological Statistics, School of Life Sciences and Biotechnology, Shanghai Jiao Tong University, Shanghai 200240, China; taimor.khaan@sjtu.edu.cn (T.K.); abbaskhan@sjtu.edu.cn (A.K.); 10 Peng Cheng Laboratory, Shenzhen 518066, China; 11 State Key Laboratory of Microbial Metabolism, Shanghai-Islamabad-Belgrade Joint Innovation Center on Antibacterial Resistances, Joint Laboratory of International Cooperation in Metabolic and Developmental Sciences, Ministry of Education and School of Life Sciences and Biotechnology, Shanghai Jiao Tong University, Shanghai 200030, China

**Keywords:** *Elizabethkingia meningoseptica*, proteome, vaccine targets, vaccines, in silico cloning

## Abstract

*Elizabethkingia meningoseptica* is a ubiquitous Gram-negative emerging pathogen that causes hospital-acquired infection in both immunocompromised and immunocompetent patients. It is a multi-drug-resistant bacterium; therefore, an effective subunit immunogenic candidate is of great interest to encounter the pathogenesis of this pathogen. A protein-wide annotation of immunogenic targets was performed to fast-track the vaccine development against this pathogen, and structural-vaccinology-assisted epitopes were predicted. Among the total proteins, only three, A0A1T3FLU2, A0A1T3INK9, and A0A1V3U124, were shortlisted, which are the essential vaccine targets and were subjected to immune epitope mapping. The linkers EAAK, AAY, and GPGPG were used to link CTL, HTL, and B-cell epitopes and an adjuvant was also added at the N-terminal to design a multi-epitope immunogenic construct (MEIC). The computationally predicted physiochemical properties of the ensemble immunogen reported a highly antigenic nature and produced multiple interactions with immune receptors. In addition, the molecular dynamics simulation confirmed stable binding and good dynamic properties. Furthermore, the computationally modeled immune response proposed that the immunogen triggered a strong immune response after several doses at different intervals. Neutralization of the antigen was observed on the 3rd day of injection. Conclusively, the immunogenic construct produces protection against *Elizabethkingia meningoseptica*; however, further immunological testing is needed to unveil its real efficacy.

## 1. Introduction

*Elizabethkingia meningoseptica*, a Gram-negative, non-motile, rod-shaped saprophytic bacterium [1] is mostly distributed in soil, plants, water, frogs, foodstuffs, and fishes. It is a hospital-acquired pathogen reported in water sources, disinfectants, and medical instruments in hospitals and can be extracted from the sputum of patients with cystic fibrosis [2]. *E. meningoseptica* has the ability to evade the host immune system and spread to other parts of host tissues. It can circulate in the bloodstream of hosts and obtain nutrients from erythrocytes [3,4,5]. Both immunocompromised and immune-competent patients are the major targets for *E. meningoseptica,* especially those frequently exposed to antibiotics, with pre-existing comorbidities and patients requiring hemodialyzers and ventilators [2,3,6,7,8,9]. The *E. meningoseptica* infection can develop into septicemia, neonatal meningitis [10,11], and neonatal sepsis [8,12,13]. Premature neonates and low-weight infants weighing less than 2500 g are especially at risk, with 84% of these cases reported to have been caused by *E. meningoseptica* at a growing mortality rate of 52% [2,7,14,15].

According to epidemiological reports, Taiwan is the emerging geographical center of *E. meningoseptica* infections over the last decade [2,3,16]. Several other reports declare Saudi Arabia, Pakistan, India, Brazil, and Kuwait to have many cases [6,17,18,19,20,21]. Treatment for *E. meningoseptica* infections should be based on the minimum inhibitory concentration (MIC) results from susceptibility tests. However, various species of *E. meningoseptica* are reported to cause resistance to multiple drugs, mainly β-lactams. The resistance is reported to be associated with different types of β-lactamases i.e., class A extended-spectrum β-lactamases and class B metallo-β-lactamases (MBLs). The Virulence Factor Database (VF) predicted 766 common virulence factors for *E. meningoseptica* in which many virulent genes were involved in the synthesis of lipo-oligosaccharides, capsule polysaccharide, catalases, proteases, peroxidase, a two-component regulatory system, superoxide dismutase, heat shock protein, and many others responsible for virulence [22,23,24]. The strain of *E. meningoseptica* posed a high level of resistivity to 16 out of 13 antibiotics, which coined it as a strong multi-drug-resistant pathogen [23,25]. The two (bla) genes encode the serine-β-lactamase blaCME and blaBlaB for eliciting cephalosporin resistance and wide-spectrum metallo-β-lactamase [26,27,28,29,30]. In the same fashion, resistance against almost 6 β-lactam medicines is provoked by five lactamase genes encoding class C β-lactamases, penicillin binding protein, metal-dependent hydrolases, β-lactamases and other β-lactamases. Moreover, *otrA*, *tetO, tetBP,* and one fluoroquinolone resistance gene, *gyrB,* could potentially incite tetracycline resistance. Additionally, for non-specific resistance, 18 multidrug resistance efflux-pumps were analyzed [23,25]

*E. meningoseptica,* is an emerging nosocomial pathogen that targets both immunocompromised and immunocompetent patients [2,3,6,7,8,9] with multidrug-resistant features [2,14,31,32,33]. To quickly address the issue of emerging pathogens, the availability of recent advanced tools can quickly highlight therapeutic options. In this regard, computational methods, i.e., understanding the resistance mechanism, greatly assisted structure-based drug designing, peptide inhibitors, and reverse vaccinology, are of great help. In the case of infectious agents, an effective subunit vaccine candidate is of great interest to encounter pathogenesis. Computational approaches, specifically immunoinformatics, can be exploited to procure antigenic proteins from the whole proteome of the target and prioritize the best potential B- and T-cell epitopes, followed by mapping of a multi-subunit peptide immunogen construct [24,34,35,36,37,38]. Hence, in this study, we employed subtractive proteomics approaches to map the protein targets with the most potential virulence in the proteome of *E*. *meningoseptica.* Next, we used reverse vaccinology tools to model a prophylactic MEIC protein against *E. meningoseptica* using CTL, HTL, and B-cell epitopes. The final MEIC candidate was then validated using molecular docking, in silico cloning, molecular dynamics simulation, and immune simulation. Our predicted MEIC demonstrated favorable physiochemical, antigenic, and non-allergenic properties. Conclusively, the MEIC was predicted to produce protection against *Elizabethkingia meningoseptica*; however, further immunological testing is needed to unveil its real efficacy.

## 2. Materials and Methods

### 2.1. Retrieval of Complete Proteome

**Figure 1:**
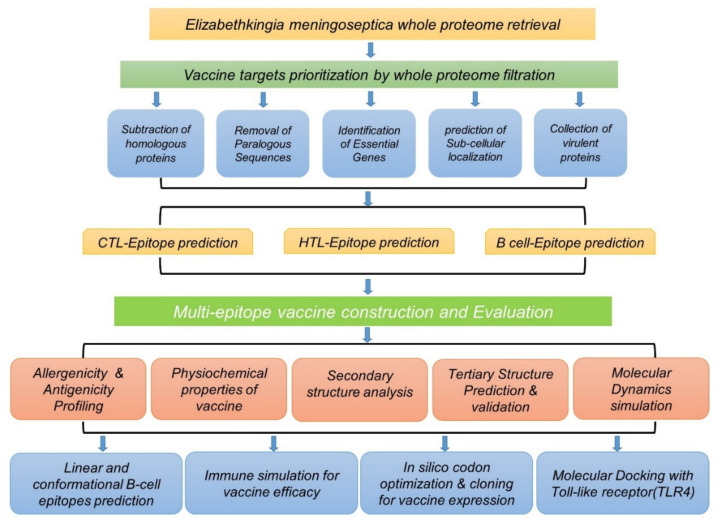
Flow chart of the multi-epitope subunit immunogenic construct against *E. meningoseptica,* following a reverse vaccinology approach.

To access the whole proteome of *Elizabethkingia meningoseptica,* we used UniProt (https://www.uniprot.org/proteomes accessed on 15 March 2021), which is a public repository to access protein-specific information [39,40]. The reference proteome, consisting of 3466 proteins in total, was retrieved using accession number UP000188947. The whole flow of the work is given in Figure 1.

### 2.2. Proteome Analysis of Elizabethkingia meningoseptica through Subtractive Proteomics

To design an immunogenic construct against any pathogen, the foremost step is the selection of proteins that are not present in the host (human) to be recognized as an antigen/foreign particle to trigger the immune system. In this approach, the first step is to remove homologous proteins from the proteome to avoid any autoimmune reaction and select pathogen-specific targets. Thus, the whole proteome retrieved from UniProt was subjected to homologous protein removal using NCBI’s BLASTp approach [41]. The whole proteome was BLAST against the reference human, considering the threshold value of 0.000001 [40], and a list of non-homologous proteins was obtained, which was then subjected to further analysis. Next, to remove the recurrence of the same sequences in the same bacterial proteome, a list of non-homologous sequences was then subjected to the CD-HIT server (http://weizhongli-lab.org/cd-hit/ accessed on 16 March 2021), using a 0.8 (80%) threshold [42]. Using the identity threshold, proteins were clustered and paralogous pairs were identified. Essential genes required for survival and pathogenesis were obtained from a list of non-paralogous proteins using the DEG (Database of Essential Genes), accessible at http://www.essentialgene.org/ (accessed on 18 March 2021) [43]. The server predicted essential genes and removed non-essential proteins from the proteome of archaea eukaryotes, which were utilized to guess the essential genes. A BLASTp approach using a 0.00001 threshold classified essential and non-essential genes [40].

### 2.3. Analysis of Subcellular Localization

Bacterial proteins that localize in the cell wall, outer membrane, periplasmic membrane, inner membrane, cytoplasm, and extracellular space play a central role in the survival, pathogenesis, and adhesion of the pathogen and are therefore considered the best targets for vaccine designing. Thus, a list of essential genes of *E. meningoseptica, obtained* from the DEG, was checked for their cellular localization using CELLO-2GO (http://cello.Life.nctu.edu.tw/cello2go/ accessed on 21 March 2021) an online webserver, to predict the subcellular localization [40].

### 2.4. Prioritizing Candidates for Potential Insilco Vaccine Construct

Previous studies suggested the use of a membrane protein for designing vaccines [44,45,46,47,48]. Therefore, in this study membrane proteins such as outer membrane, cell membrane, extracellular, and peripheral membrane proteins were used to develop a subunit peptide immunogenic construct.

### 2.5. Collection of Virulent Proteins

The virulence factor database (VFDB) is a repository of virulence [49]. Membrane proteins of *E. meningoseptica* were subjected to BLASTp for obtaining the virulent proteins for designing in silico immunogenic construct predictions. The best virulent proteins, based on their antigenicity, toxicity, allergenicity, molecular weight, and length, were processed for computational immunogenic construct designing.

### 2.6. Predictions of Epitopes

CTL, B-cell and HTL epitopes were predicted with the help of NETCTL 1.2 (http://www.cbs.dtu.dk/services/netctl/ accessed on 22 March 2021), ABCPred (http://crdd.osdd.net/raghava/abcpred/ accessed on 22 March 2021), and IEDB MHC II server (http://www.iedb.org/ accessed on 22 March 2021), respectively [36,45,46,47]. The threshold for CTL epitope prediction was set at 0.75, whereas for B-cell epitope prediction, specificity was kept to 75% at a 0.51 threshold [48]. HTL epitopes were predicted for five human HLA alleles (DRB1*01:01, DRB1*01:02, DRB1*01:03, DRB1*01:04, and DRB1*01:05). An IC50 score was used to check the binding affinity of the peptide with receptor. An IC50 score of <50 nm, <500 nm, and <5000 nm indicated greater, intermediate, and low binding affinities, respectively. The epitopes with an IC50 score of <50 nm were selected for immunogenic construct designing.

### 2.7. Multiepitope Vaccine Construction

The predicted CTL, HTL, and B-cell epitopes were joined together with the help of AAY and GPGPG linkers. CTL epitopes were linked with AAY linkers, whereas a GPGPG linker was used to join the last CTL epitope with HTL as well as to link HTL and B-Cell epitopes. Human beta-defensin 3 (1KJ6_A) chain A was joined with CTL epitopes with EAAAK linker for the enhancement of immunogenicity [36,50]. These linkers helped the epitopes to produce suitable sites for binding to TAP transporter, increased the immunogens stability, and enhanced epitope presentation while the GPGPG linker stimulated HTL responses and conserved conformational dependent immunogenicity of helpers as well as antibody epitopes.

### 2.8. Evaluation of Vaccine Construct

#### 2.8.1. Prediction of Allergenicity and Antigenicity

The AlgPred (http://www.imtech.res.in/raghava/algpred/ accessed on 26 March 2021) and AllerTOP v. 2.0. (https://www.ddg-pharmfac.net/AllerTOP/ accessed on 26 March 2021) servers were used to check the non-allergenic nature of the immunogenic construct at a 0.4 threshold. The antigenicity of the immunogenic construct was determined by the VaxiJen server (http://www.ddg-pharmfac.net/vaxijen/vaxijen.html accessed on 26 March 2021) at a 0.4 threshold [36,50,51].

#### 2.8.2. Physiochemical Property Calculations

The computationally predicted physiochemical properties of the immunogenic construct were calculated by using the ProtParam (https://web.expasy.org/protparam/, accessed on 27 March 2021) tool. Various properties such as the molecular weight, atomic composition, theoretical PI, amino acid composition, half-life in *E. coli*, and aliphatic index were calculated. The extinction coefficient, GRAVY (grand average of hydropathicity), and instability were checked [51].

#### 2.8.3. Tertiary Structure Prediction, Visualization, and Refinement

The 3D structure of the immunogenic construct was modeled using the Robetta server for predictions. Robetta incorporated comparative, domain-based and ab initio modeling for a reliable 3D structure. PyMOL 1.7.1. (Schrödinger, Inc., Boston, MA, USA) was used for the visualization of the immunogen’s tertiary structure [36,50]. The Galaxy Refine (http://galaxy.Seoklab.org/ accessed on 1 April 2021) tool was used for the refinement of the tertiary structure, considering both local and global quality [51].

#### 2.8.4. Validation of the Tertiary Structure

The ERRAT (http://services.mbi.ucla.edu/ERRAT/ accessed on 1 April 2021), PROSA-web (https://prosa.Servi-ces.came.sbg.acat/prosa.php accessed on 1 April 2021), PROCHECK (https://servicesn.mbi.ucla.edu/PROCHECK/ accessed on 1 April 2021), and Verify 3D (https://servicesn.mbi.ucla.edu/Verify3D/ accessed on 1 April 2021) tools were used for the validation of the immunogen’s tertiary structure [51].

#### 2.8.5. Mapping of the B-Cell Epitopes in the Designed Vaccine’s Tertiary Structure

B-cell epitopes (linear and conformational) are the antigenic determinants of proteins. However, antibody production is high against conformational B-cell epitopes. Therefore, it is considered to be antigenic determined and is preferred for immunogen designing [47]. Ellipro (http://tools.iedb.org/ellipro/, accessed on 5 April 2021), an online tool [48], was used for the prediction of the B-cell epitopes. Ellipro assigned a protrusion index (PI) score and made clusters on the basis of distance, R, measured in (Å). A greater R-value was the indication of higher discontinuous epitopes [36,50,51].

#### 2.8.6. Molecular Dynamics (MD) Simulations

Molecular dynamics simulations were performed to check the dynamic stability of the immunogenic construct [45]. This simulation was performed on the AMBER 20 simulation package (University of California, Los Angeles, CA, USA) [47]. Different steps such as topology generation, structure preparation, solvation, and neutralization with ions were performed before the MD simulation. The Waterbox cutoff radius was set at 10.0 Å, and the minimization step was followed by heating, equilibrium, and production. After an MD simulation of 20 ns, the obtained graphs were assessed for stability by calculating the RMSD and RMSF. The simulations were performed at the “Centre for High-Performance Computing, Shanghai Jiao Tong University” using a DGX2 cluster (SJTU, Shanghai, China) and took 30 h to complete.

#### 2.8.7. Immune Simulation for Vaccine Efficacy

The C-ImmSim server (http://150.146.2.1/C-IMMSIM/index.php, accessed on 15 April 2021) was applied to predict the human immune response against the immunogenic construct [52]. For obtaining the response, the FASTA file of the immunogenic construct with the Human beta-defensin 3 adjuvant was uploaded to the server, and the resultant graphs show the secretions of various immune cells. The Gram-ve bacterial model was used for the simulation with the number of antigens to inject set at 1000 (default).

#### 2.8.8. Molecular Docking of Our Ligand with TLR4

The TLR4 immune receptor was selected for docking with the immunogenic construct because it can trigger cytokine overproduction and can upregulate the TSPO-associated proteins [49,50,51]. The human TLR4 structure (homodimer form) was obtained from the RCSB with PDB ID: 3FXI. This structure consists of chains A and B, containing 605 amino acids each [52]. The ClusPro 2.0 server (Stony Brook, Long Island, NY, USA) was used for the docking of the TLR4 homodimer with the immunogenic construct. This process was completed in three steps; the first structure was refined, followed by energy minimization and rigid body docking. The best TLR4 and immunogenic construct complex was selected based on lower energy scores and the best visualization.

#### 2.8.9. In Silico Codon Optimization and Cloning of the Vaccine Construct

JCat (http://www.mrc-lmb.cam.ac.uk/ms/methods/codon.html, accessed on 21 April 2021) was used for the reverse translation process and the optimization of codons to gain the highest expression in *E. coli* using computational algorithms. The highest expression was assured by the Jcat tool. The GC contents percentage and CAI scores of the immunogenic construct were recorded. It was then cloned into PET-61-DEST (+) plasmid. Appropriate restriction enzymes were added to cut the plasmid and the immunogen at the particular parts. The immunogen was performed by the SnapGene tool (GSL Biotech, Evanston, IL, USA).

## 3. Results and Discussion

### 3.1. Whole-Proteome Sequence Retrieval

The whole proteome of *E. meningoseptica* was retrieved from UniProt using the proteome ID (UP000188947) with a total of 3466 proteins, out of which the potential protein targets were prioritized to design a MEIC against *E. meningoseptica.*

### 3.2. Removal of Homologous Protein Sequences

The BLASTp against the human proteome resulted in homologous protein sequences consisting of an aligned file of 1111 protein sequences. After discarding the homologous proteins, a total of 2355 non-homologous proteins were obtained for further analysis.

### 3.3. Removal of Paralogous Sequences

By utilizing the CD-HIT server, among the total of 2355 non-homologous proteins, only seven paralogous protein sequences, at a 0.8 threshold, were identified. This resulted in 2348 shortlisted non-homologous and non-paralogous proteins.

### 3.4. Essential Gene Identification

Subtractive proteomics approaches used to find targets for vaccine designs mainly rely on finding essential proteins required for pathogen survival and pathogenesis. Next, a total of 1115 essential proteins with sizes >100 amino acids were identified through the utility of BLASTp against the DEG database. This step revealed the protein crucial for pathogen survival, leading the way to the subsequent steps. On the other hand, proteins with sizes <100 amino acids were excluded because of their minimal importance in immunogenic construct designing.

### 3.5. Analysis of Sub-Cellular Localization

It is also important to find the sub-cellular localization of proteins to find suitable vaccine targets. The total (1115) essential proteins identified for *E. meningoseptica* were further evaluated through CELLO-2GO (http://cello.life.nctu.edu.tw/cello2go/ accessed on 21 March 2021). The results revealed a total of 752 cytoplasmic proteins and 363 membrane proteins as potential drug and vaccine targets, respectively. Herein, only shortlisted membrane proteins were selected for further investigation. The total (363) shortlisted membrane proteins comprised 24 extracellular, 146 inner membrane, 109 outer membrane, and 84 periplasmic proteins.

### 3.6. Virulent Protein Collection

The identification of virulent proteins is also essential in shortlisting target proteins in immunogenic construct designing. All of the shortlisted membrane proteins of *E. meningoseptica* were further evaluated individually through PSI/PHI BLASTp with a threshold score of 0.002. Subsequently, proteins having >30% sequence identity and a bit score of >100 were considered as virulent candidates. Only one virulent protein was obtained among the extracellular proteins. Of the 146 inner membrane proteins, 10 fell in the criteria for virulence. Outer membrane proteins resulted in 9 out of 109 as virulent, followed by 8 out of 84 periplasmic proteins. Hence, a sum of 28 proteins was finalized as key mediators in the virulence of the pathogen.

### 3.7. Prioritization of Proteins for Final Vaccine Designing and Their Physiochemical Parameters

It is also pivotal to prioritize highly immunogenic proteins for inclusion in the final immunogenic construct design. Based on different parameters, including high antigenicity, non-allergenicity (bit score), high virulence, optimal molecular weight, values of sequence identity, and suitable length of amino acid, only 3 proteins out of the 28 best candidate proteins were prioritized as target proteins for immunogenic construct designing. The three finalized proteins A0A1T3FLU2, A0A1T3INK9, and A0A1V3U124 included one periplasmic protein and two outer membrane proteins, respectively. To depict numerous crucial parameters suitable for immunogen designing, including molecular weight determination, the ExPasy server (https://www.bioinformatics.org/sms/protmw.html accessed on 21 October 2021) was utilized. Based on the calculated protein sizes, those with a higher number of amino acids were preferred as this increases the chances of epitope prediction. Similarly, higher antigenic proteins were exploited for further analysis as they provoke immunity to a greater extent. Moreover, the allergenic/non-allergenic nature of proteins was evaluated to exclude allergenic proteins. The shortlisted proteins having molecular weights <110 kg Daltons and lengths >100 amino acids were selected for immunogen construction. The three finalized proteins with optimal properties for inclusion in the immunogen designs are demonstrated in (Table 1).

### 3.8. Antigenic Epitope Prediction and Prioritization

T-cell and B-cell epitope identification is a prerequisite in multi-epitope-based immunogen designs against infectious pathogens. NetCTL 1.2. predicted different numbers of CTL epitopes for each protein. Out of all CTL epitopes, two high-scoring CTL epitopes for each protein were finalized based on high combined scores as shown in Table 2. The IEDB server was accessed, which resulted in thousands of HTL epitopes for each protein. Among these, a total three low percentile rank, non-allergenic and non-overlapping HTL epitopes were selected for vaccine design as shown in (Table 3). On the other hand, a total number of 12 continuous B-cell epitopes, comprising 196 amino acids with a score range from 0.537 to 0.811, were predicted using the Ellipro tool. Moreover, a total of 215 amino acids containing 6 discontinuous B-cell epitopes with a score range from 0.519 to 0.692 were also predicted. Among these, two high-scoring BL epitopes for the outer membrane proteins and two BL epitopes from the periplasmic protein were finalized, as shown in (Table 4).

### 3.9. Multi-Epitope Immunogen Construction

**Figure 2:**
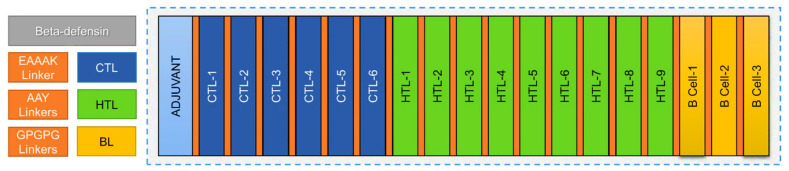
The schematic representation of the multi-epitope-based immunogen construct against *E. meningoseptica.* A total of 18 immune epitopes, including 6 CTL (blue color) joined by AAY linkers (orange color), 9 HTL (green color) joined by GPGPG linkers (orange color), and 3 BL epitopes (yellow color) joined by GPGPG linkers (orange color), from the selected three proteins (A0A1T3INK9, A0A1V3U124, and A0A1T3FLU2) are shown. The N-terminal-linked adjuvant Human Beta-defensin 3 (cyan color) attached with an EAAK linker (orange color) is also shown.

The final immunogen construct was designed by exploiting the putative immune epitopes identified for each prioritized protein. The designed immunogenic construct consisted of an N-terminal-linked adjuvant called Human Beta-defensin 3 (GIINTLQKYYCRVRGGRCAVLSCLPKEEQIGKCSTRG RKCCRRKK) followed by the attachment six CTL epitopes, nine HTL epitopes, and three BL epitopes. A total of 18 epitopes were linked, resulting in a construct size of 362 amino acids. The CTL epitopes were fused using AAY linkers, while the HTL and B-cell epitopes were joined through GPGPG linkers, as shown in (Figure 2).

### 3.10. Immunogen Property Evaluation

#### 3.10.1. Allergenicity and Antigenicity Status Evaluation

It is vital to check the allergenicity and antigenicity status of an immunogenic construct to ensure the production of a robust immune response and avoid allergy-related complexities associated with immunogen designs. AlgPred (http://www.imtech.res.in/raghava/algpred/ accessed on 21 October 2021) and VaxiJen (http://www.ddg-pharmfac.net/vaxijen/vaxijen.html accessed on 21 October 2021) were utilized to evaluate the allergenicity and antigenicity status of the vaccine construct. Herein, the allergenicity score of −0.40382219 at a threshold of −0.4 and antigenicity score 0.7538 at a threshold of 0.4 were obtained. Moreover, this validated the immunogenic status of the vaccine construct.

#### 3.10.2. Identification of Physiochemical Properties

The prediction and evaluation of physiochemical parameters helps to inquire about the suitability of the immunogenic construct in further experimental designs. Herein, the physiochemical properties of the immunogenic construct were also evaluated through the use of the ProtParam (https://web.expasy.org/protparam/ accessed on 21 October 2021) and PEPS-TATS (https://www.ebi.ac.uk/Tools/seqstats/embosspepstats/ accessed on 21 October 2021) web servers. This predicted the significant physiochemical properties of the immunogenic construct, including high stability, a hydrophilic nature, a higher aliphatic index (67.96), and a Theoretical pI (9.20). The chemical formula (C1678H2616N482O506S12) generated for the designed immunogenic comprised a total of 5294 atoms. Similarly, the molecular weight calculated was 38 KDa while a greater half-life of the immunogen construct in *E. coli* (>10 h), yeast (>20 h) and mammalian reticulocytes (>30 h) was found. These results further validated our immunogen construct to be stable and possess other suitable parameters.

#### 3.10.3. Secondary and Tertiary Structure Predictions and Analysis

**Figure 3:**
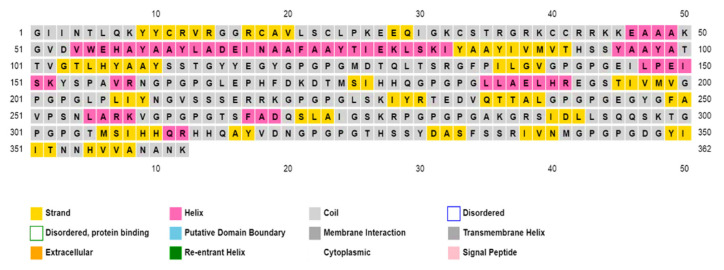
Distribution of secondary structure elements of the proposed immunogenic construct. Alpha-helices are shown as magenta, coils as grey, and beta sheets as yellow color. The other regions were not predicted as a part of immunogen construct.

The structural characterization, including the secondary and tertiary structure prediction, helped to understand the composition of the immunogen. The secondary structures of the final immunogen construct based on amino acid sequences were predicted through the utility of PSIPRED (http://bioinf.cs.ucl.ac.uk/psipred/ accessed on 21 October 2021), as illustrated in (Figure 3). Additionally, the *SOPMA* server (https://npsa-prabi.ibcp.fr/NPSA/npsa_sopma.html accessed on 21 October 2021) was utilized to generate the percent distribution of the alpha-helices (19.61%), random coils (57.46%), and beta sheets (22.93%). To show all domains, an image was also obtained by the PDBsum database.

#### 3.10.4. Modeling of the 3D Structure of MEIC

**Figure 4:**
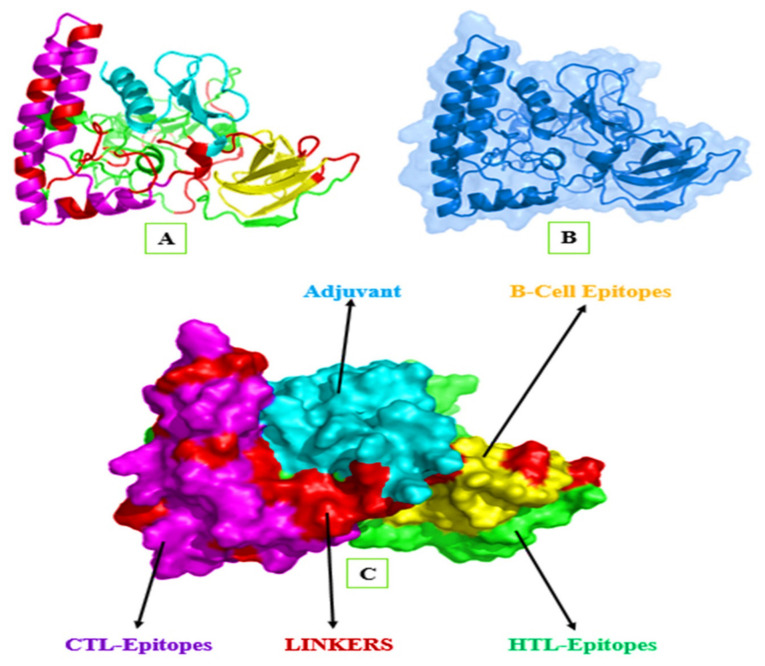
Visualization of the 3D structure of the proposed immunogen. Panel (**A**) multi-epitope immunogen presented in distinctive colors, where (cyan color) shows adjuvant, (magenta color) shows CTL epitopes, (green color) shows HTL epitopes, (yellow color) shows BL epitopes, and (red color) shows the different linkers (AAY, GPGPG, and EAAAK). Moreover, in Panel (**B**) (marine color) shows a cartoon representation with 80% surface transparency, while Panel (**C**) shows the complete (100%) surface shape of the multi-epitope immunogen subunits, indicated by different colors.

The Robetta server was utilized for 3D structure generation using multiple templates. The predicted structure was validated through different online servers, including ProSA-web, ERRAT, verify 3D, and PROCHECK, leading to the selection of model number-1 out of the five models. The final validated model was visualized through the PyMOL 1.7.1. software as shown in (Figure 4).

#### 3.10.5. Refinement of Tertiary Structure

The GalaxyRefine server generated five refined structures based on poor rotamers, Rama favored, mol Probity, GDT-HA, Clash score, and RMSD. The higher score obtained for GDT-HA Rama favored was considered as best, whereas low scores for poor rotamers, mol Probity, and RMSD were considered favorable. The resulting finalized model structures are shown in (Table 5).

#### 3.10.6. Validation of the Tertiary Structure

**Figure 5:**
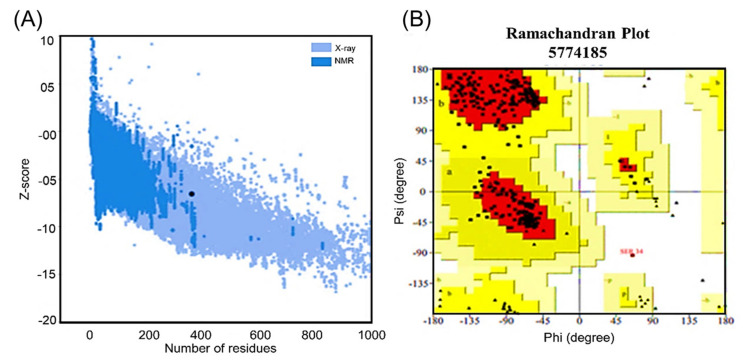
Analysis of the Ramachandran (PROCHECK) and PROSA-web servers. Panel (**A**) shows the ProSA-web analysis with the depicted Z score (−6.54) indicating a valid 3D structure. Panel (**B**) shows the Ramachandran plot with the amino acid distribution in the allowed and disallowed regions and favored regions. It also shows the higher percentage (92.4%) of residues in the favored region, followed by the lower percentage (7.3%) of residues in the allowed region, and the lowest percentage (0.4%) reported in the disallowed region before the MD simulation.

The validation of tertiary structures is an important step in peptide-based immunogen designs. The final 3D structure of the immunogen design was validated through the utility of four different servers. Firstly, ProSA-web (https://prosa.services.came.sbg.ac.at/prosa.php accessed on 21 October 2021) was used to predict the Z score (−6.54), as shown in (Figure 5A). Secondly, a quality factor of 88.8 was obtained through ERRAT (https://servicesn.mbi.ucla.edu/ERRAT/ accessed on 21 October 2021) during validation analysis. Moreover, the structure was also validated through the use of an online server (https://servicesn.mbi.ucla.edu/Verify3D/ accessed on 21 October 2021) which showed 88.95% of the residues with an average 3D–1D score of ≥0.2. Moreover, a higher percentage (92.4%) of residues were found in the favored region. This analysis was performed using the PROCHECK web tool (https://servicesn.mbi.ucla.edu/PROCHECK/ accessed on 21 October 2021) which revealed 7.3% within the allowed regions and only 0.4% of the residue in other regions via Ramachandran plot. The serine residue, number 34, appeared in the disallowed region as given in (Figure 5B). Herein, model 1 was finalized after an average-scores-based validation for further analysis.

#### 3.10.7. Tertiary Immunogen Structure Validation through Molecular Dynamics (MD) Simulation

**Figure 6:**
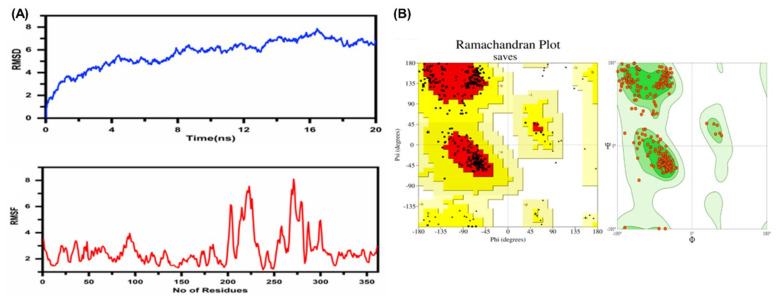
(**A**) The RMSD and RMSF graphs obtained during the molecular dynamics simulation (MD) analysis of the immunogen design. The plots maintained an average flexibility of 2.5 Å with a maximum of 7.0 Å in the highly flexible section. (**B**) Post-simulation Ramachandran graph.

MD simulation helps us to understand the correct binding confirmations and protein folding. The RMSD and RMSF graphs were obtained for the immunogen construct after molecular dynamics simulations. The stability of the vaccine was observed as a result of the RMSD graph. Initially, the RMSD increased but it attained equilibration later. The RMSD of the 3D vaccine structure was observed to be flattened until 20 ns and stabilized at 17 ns. The convergence remained minimal and the average RMSD was found to be 4.0 Å. The flexibility of the residues was evaluated during the RMSF analysis, which showed a lower flexibility throughout the procedure. However, a higher flexibility was observed for the region between 200 and 238 and 264 and288 due to the presence of the loop region, as given in Figure 6A. These values confirmed the stability of our 3D vaccine. These MD analyses were further validated through the Verify 3D and PROCHECK servers which showed improved 3D structures. Herein, the Ramachandran plot showed a higher percentage (81.1%) of residues in the favored region, followed by 18.9% and 0.0% in the allowed and disallowed regions, respectively, as shown in (Figure 6B). This resulting MD-simulated 3D structure, with increased residues in the allowed region and decreased residues in the disallowed region, was then forwarded to docking analysis. Before and after the MD simulation, these results were verified with the Swiss-model utility (https://swissmodel.expasy.org/assess accessed on 21 October 2021) for the obtained structures.

#### 3.10.8. Analysis of Immunogenic Construct Interaction with TLR’s

**Figure 7:**
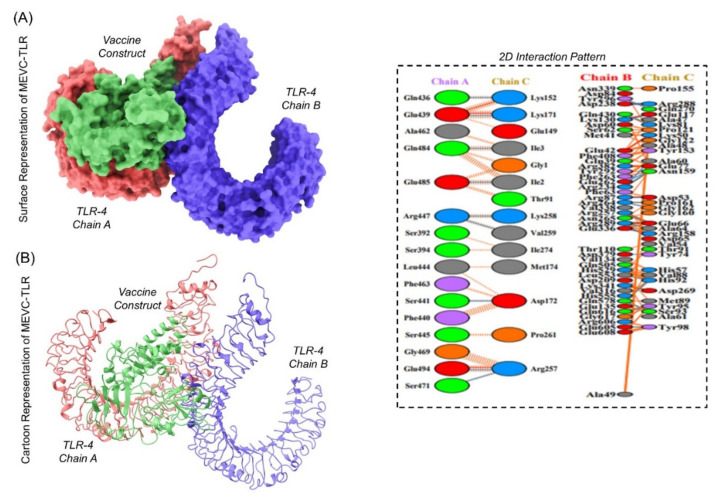
The docked complex of the constructed immunogen and the TLR4 homodimer receptor with A and B Chains. Whereas (blue color) represents the B-chain of TLR4, (orange color) represents the A-chain of TLR4, while (green color) represents the designed immunogen in all the of the representative images. (**A**) Surface representation of the docked complex. (**B**) Cartoon shape and the front side of the docked complex. The right panel represents the 2D interaction pattern of the docked complex.

The interaction analysis of the designed immunogen with human TLR receptors helps to understand the binding affinity involved in complex formation. The *ClusPro* web server (https://cluspro.bu.edu/ accessed on 21 October 2021) generated a total of 30 clusters for the vaccine and receptor complexes. Among these 30 models, an appropriate selection of docked models was performed after visualization at different angles (Figure 7) by exploiting the PyMOL *1.7.1* software. The lowest energy (−1193.9) and total energy (−1059.3) scores were also obtained for the finalized model.

#### 3.10.9. Codon Optimization and In Silico Cloning of the Final Immunogen

**Figure 8:**
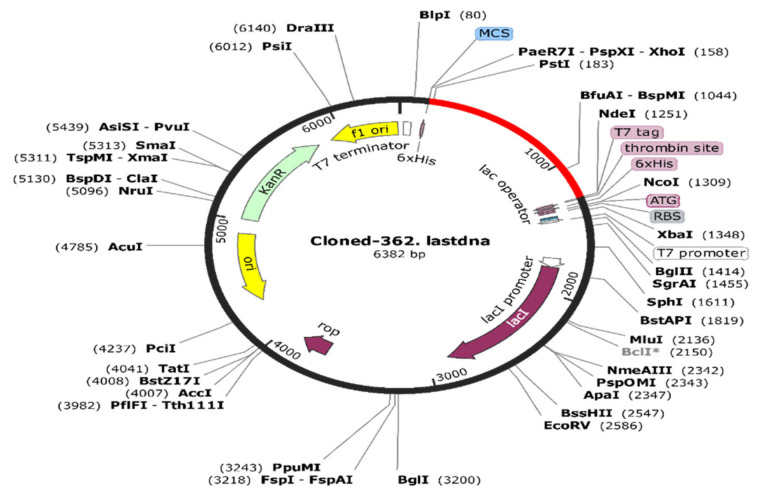
In silico cloning. The inserted optimized DNA sequences of proposed immunogenic construct in plasmid vector map pET-28a (+) with restriction sites of six nucleotides, XhoI (158) and NdeI (1251). The insert sequence is denoted by the red color while vector is denoted by the black color in the circular plasmids.

It is also vital to obtain an optimized DNA sequence for an immunogenic construct that can be cloned into an expression vector subjected to protein purification. Firstly, the Jcat server (http://www.mrc-lmb.cam.ac.uk/ms/methods/codon.html accessed on 21 October 2021) was employed for in silico codon optimization. This involved codon adaptation for the desired expression system by choosing the E. coli strain K12 as a host organism. Similarly, the obtained codon adaptation index (CAI), 0.982, and guanine-cytosine (GC) content value, 54.69%, were found to be favorable. Next, the SnapGene software v3.3.4 was utilized for the insertion of the optimized DNA sequence through the selection of suitable restriction enzymes, including the N-terminal CTCGAG (Xho1) and C-terminal CATATG (Nde1). SnapGene also confirmed the absence of other restriction enzymes in both the vector backbone and the vaccine construct. Finally, the optimized DNA sequences were inserted in a pET-28a (+) vector having a length of 5.4 kbp. The immunogenic construct sequence consisted at both ends of 6-histidine tag residues within the vector required in protein purification. The total size of the newly constructed plasmid vector was 6.38 kbps, as shown in (Figure 8).

#### 3.10.10. Immune Simulation for Immunogenic Efficacy Prediction

**Figure 9:**
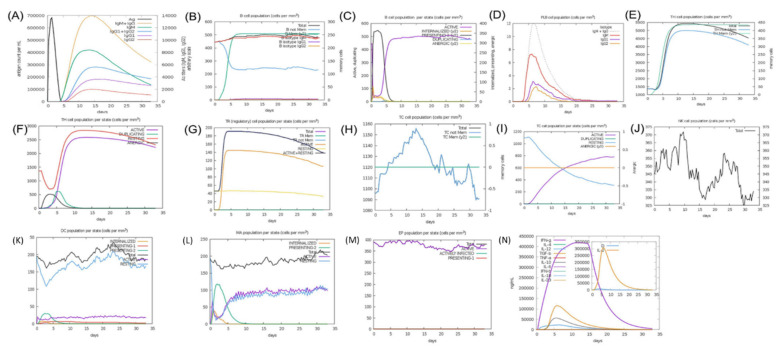
In silico immune simulations. The potential induction of immune response factors upon injection of the constructed immunogen as an antigen. (**A**) shows the total antigen and immunoglobulin counts, (**B**) shows the total (per mm^3^) B-cell production, (**C**) shows the levels of B-cell lymphocyte production, (**D**) shows production of Plasma B lymphocytes, (**E**) shows the total (per mm^3^) of CD4 T-helper lymphocytes produced, (**F**) shows the total CD4 T-helper lymphocytes produced, (**G**) represents the CD4 T-regulatory lymphocyte production, (**H**) represents the CD8 T-cytotoxic lymphocyte production, (**I**) represents the CD8 T-cytotoxic lymphocyte production, (**J**) represents natural killer cell production, (**K**) represents dendritic cell production, (**L**) represents macrophages produced, (**M**) represents epithelial cells produced, and (**N**) represents the production of immune specific IFN-g and IL-2 (Cytokines) factors.

Immune simulation analysis helps to predict the antigenic potential of a designed immunogenic in producing an immune response. This involves the quantification of antibody titers produced against a selected antigen in a specified time. The C-ImmSim is an online server (http://150.146.2.1/C-IMMSIM/index.php accessed on 21 October 2021) with default parameter generated graphs representing the different antibody responses (Figure 9). The production of IgM and IgG against the antigen started from day 5 and reached the highest levels on day 15 (panel A). Similarly, the robust immune response was observed from B-cell isotypes, indicated by the high production of IgM and memory cell formation since day 1 after injection, with a gradual increase until day 5, followed by uniformity until the end (panel B). Similarly, the B lymphocyte (B cell) production (per mm^3^) was observed, reaching the highest peaks on day 5 (panel C). Moreover, Plasma B lymphocyte (PLB) cell production was observed from day 3 (panel D).

The production of CD4 T-helper lymphocytes (TH) and memory cells attained the highest values on day 5 (panel E). The production of CD8 T-cytotoxic lymphocytes and memory cell counts are also shown in (panel H and I). Natural Killer cells were found to increase their intensity, specifically from days 5 to 25. The levels of dendritic cells (DC) was also observed in the (panel H) curves. Consequently, high values for macrophages and epithelial cells and increased levels of IFN-g and IL-2 (Cytokines) production were also witnessed in the graph. (Panel L, M and N).

## 4. Conclusions

This present scientific study used subtractive proteomics and an immunoinformatics pipeline to design an immunogenic construct from the proteome of *Elizabethkingia meningoseptica* for the control of nosocomial infection. Such an immunogen is capable of providing immunity for both types of immune systems, as predicted computationally. Several approaches were employed, such as molecular docking, simulation for thermal stability, and in silico cloning, for validation of its thermal stability inside the human body, its better expressions in vectors for its mass production, and the inability to trigger an immune response. The designed immunogenic candidate should be tested in vitro and in vivo for final use.

## Figures and Tables

**Table 1:** The three whole-proteome-based shortlisted proteins of *E. meningoseptica* with optimal properties for inclusion in immunogen designs. Furthermore, the antigenic and non-allergenic nature of the proteins is also demonstrated.

Accession/Cellular Location	Sequence Length	Bit-Sore	Percent Identity	Molecular Weight	Antigenicity Value	Allergenicity
A0A1T3FLU2	207aa	158 bits	40%	23.32 kDa	0.5802	Non-allergenic
A0A1T3INK9	243aa	127 bits	36%	27.48 kDa	0.5383	Non-allergenic
A0A1V3U124	505aa	237 bits	37%	54.29 kDa	0.5170	Non-allergenic

**Table 2:** The cytotoxic T lymphocyte epitopes predicted for each of the selected proteins of *E. meningoseptica,* including amino acid sequence, amino acids length, and other parameters.

Cellular Location/Accession	Type of Epitope	Epitope Sequence	Epitope Length	(MHC) Binding Affinity	Combined Score
periplasmic (A0A1T3FLU)	CTL	GVDVWEHAY	9	0.5794	2.7407
	CTL	LADEINAAF	9	0.2387	1.2355
outer membrane (A0A1T3INK9)	CTL	TIEKLSKIY	9	0.2651	1.4033
	CTL	IVMVTHSSY	9	0.1531	0.9540
outer membrane (A0A1V3U124)	CTL	ATTVGTLHY	9	0.6437	3.0377
	CTL	SSTGYYEGY	9	0.4328	2.1323

**Table 3:** The helper T-cell epitope predicted for each of the selected proteins of *E. meningoseptica* with information, including their peptide length, percentile rank, position, and important parameters.

Cellular Location/Accession	HTL S. No	Type of Allele	Epitope Sequence	Start-End	Percentile Ranks	Allergenicity Values
periplasmic (A0A1T3FLU)	1st	HLA-DRB3*02:02	MDTQLTSRGFPILGV	151–165	22	−1.068
	2nd	HLA-DRB3*02:02	EILPEISKYSPAVRN	56–70	33	−0.567
	3rd	HLA-DRB1*03:01	LEPHFDKDTMSIHHQ	16–30	55	−0.521
outer membrane (A0A1T3INK9)	1st	HLA-DRB3*02:02	LPLIYNGVSSSERRK	106–120	0.75	−0.720
	2nd	HLA-DRB1*07:01	LLAELHREGSTIVMV	183–197	14	−0.898
	3rd	HLA-DRB3*01:01	LSKIYRTEDVQTTAL	7–21	9.7	−0.658
outer membrane (A0A1V3U124)	1st	HLA-DRB5*01:01	EGYGFAVPSNLARKV	272–286	6.9	−0.886
	2nd	HLA-DRB3*01:01	TSFADQSLAIGSKRP	362–376	28	−0.591
	3rd	HLA-DRB4*01:01	AKGRSIDLLSQQSKT	215–229	4.1	−0.542

**Table 4:** The B-cell epitopes predicted for each of the selected proteins of *E. meningoseptica* including sequence information, position, and other parameters.

Cellular Location/Accession	Type of Epitope	Epitope Sequence	Position	aa Length	Combined Scores
periplasmic (A0A1T3FLU)	B-cell	TMSIHHQRHHQAYVDN	24	16	0.96
	B-cell	ATANQDNPLMDTQLTS	142	16	0.92
outer membrane (A0A1T3INK9)	B-cell	THSSYDASFSSRIVNM	198	16	0.88
	B-cell	EIMEKINIKHRAKHYP	124	16	0.88
outer membrane (A0A1V3U124)	B-cell	DGYIITNNHVVANANK	129	16	0.96
	B-cell	DVVKVTYLRNGKESTT	378	16	0.91

**Table 5:** The selection of an initial model for further analysis was based on obtained scores in comparison to the other five models generated by GalaxyRefine server. This was also reflected by the lower Rama favored score obtained for the initial model with little deviation in comparison to other five models. Similarly, higher scores in other parameter let us make the final selection of initial model for validation.

Models	GDT-HAs	RMSDs	Mol-Probity	Clash Scores	Poor Rotamers	Rama Favored
Initial model	1.0000	0.000	1.175	3.9	0.0	98.1
MODEL 1	0.9883	0.318	1.690	15.4	0.7	98.3
MODEL 2	0.9779	0.336	1.639	13.5	0.7	98.3
MODEL 3	0.9855	0.332	1.644	13.7	0.7	98.3
MODEL 4	0.9807	0.338	1.639	13.5	0.4	98.3
MODEL 5	0.9786	0.357	1.709	16.1	0.4	98.3

## References

[1] Sztajnbok J., Troster E. (1998). Community-acquired *Chryseobacterium meningosepticum* pneumonia and sepsis in a previously healthy child. J. Infect..

[2] Hsu M.-S., Liao C.-H., Huang Y.-T., Liu C.-Y., Yang C.-J., Kao K.-L., Hsueh P.-R. (2011). Clinical features, antimicrobial susceptibilities, and outcomes of *Elizabethkingia meningoseptica* (*Chryseobacterium meningosepticum*) bacteremia at a medical center in Taiwan, 1999–2006. Eur. J. Clin. Microbiol. Infect. Dis..

[3] Jean S., Lee W., Chen F., Ou T.Y., Hsueh P. (2014). *Elizabethkingia meningoseptica*: An important emerging pathogen causing healthcare-associated infections. J. Hosp. Infect..

[4] Breurec S., Criscuolo A., Diancourt L., Rendueles O., Vandenbogaert M., Passet V., Caro V., Rocha E.P., Touchon M., Brisse S. (2016). Genomic epidemiology and global diversity of the emerging bacterial pathogen *Elizabethkingia anophelis*. Sci. Rep..

[5] Lau S.K., Chow W.-N., Foo C.-H., Curreem S.O., Lo G.C.-S., Teng J.L., Chen J.H., Ng R.H., Wu A.K., Cheung I.Y. (2016). *Elizabethkingia anophelis* bacteremia is associated with clinically significant infections and high mortality. Sci. Rep..

[6] Tuon F.F., Campos L., de Almeida G.D., Gryschek R.C. (2007). *Chryseobacterium meningosepticum* as a cause of cellulitis and sepsis in an immunocompetent patient. J. Med. Microbiol..

[7] Lin Y.-T., Chiu C.-H., Chan Y.-J., Lin M.-L., Yu K.-W., Wang F.-D., Liu C.-Y. (2009). Clinical and microbiological analysis of *Elizabethkingia meningoseptica* bacteremia in adult patients in Taiwan. Scand. J. Infect. Dis..

[8] Chiu C.-H., Waddingdon M., Greenberg D., Schreckenberger P.C., Carnahan A. (2000). Atypical *Chryseobacterium meningosepticum* and meningitis and sepsis in newborns and the immunocompromised, Taiwan. Emerg. Infect. Dis..

[9] Lin P.-Y., Chu C., Su L.-H., Huang C.-T., Chang W.-Y., Chiu C.-H. (2004). Clinical and microbiological analysis of bloodstream infections caused by *Chryseobacterium meningosepticum* in nonneonatal patients. J. Clin. Microbiol..

[10] Ceyhan M., Yıldırım I., Tekelı A., Yurdakok M., Us E., Altun B., Kutluk T., Cengiz A.B., Gurbuz V., Barın C. (2008). A *Chryseobacterium meningosepticum* outbreak observed in 3 clusters involving both neonatal and non-neonatal pediatric patients. Am. J. Infect. Control.

[11] Lambiase A., Del Pezzo M., Raia V., Sepe A., Ferri P., Rossano F. (2007). *Chryseobacterium* respiratory tract infections in patients with cystic fibrosis. J. Infect..

[12] HY C.C., Chiu N.-C., Li W.-C., Huang F.-Y. (2000). Characteristics of neonatal bacterial meningitis in a teaching hospital in Taiwan from 1984–1997. J. Microbiol. Immunol. Infect. Wei Mian Yu Gan Ran Za Zhi.

[13] Joshi P., Shah B., Joshi V., Kumar A., Singhal T. (2019). Treatment of *Elizabethkingia meningoseptica* Neonatal Meningitis with Combination Systemic and Intraventricular Therapy. Indian J. Pediatrics.

[14] Steinberg J.P. (2009). Other gram-negative and gram-variable bacilli. Mandell, Douglas, and Bennett’s Principles and Practice of Infectious Diseases.

[15] Issack M.I., Neetoo Y. (2011). An outbreak of *Elizabethkingia meningoseptica* neonatal meningitis in Mauritius. J. Infect. Dev. Ctries..

[16] Sun G., Yu X., Bao C., Wang L., Li M., Gan J., Qu D., Ma J., Chen L. (2015). Identification and characterization of a novel prokaryotic peptide: N-glycosidase from *Elizabethkingia meningoseptica*. J. Biol. Chem..

[17] Barnawi A.I., Kordy F.N., Almuwallad O.K., Kassarah K.A. (2020). Early neonatal sepsis and meningitis caused by *Elizabethkingia meningoseptica* in Saudi Arabia. Saudi Med. J..

[18] Umair A., Nasir N. (2020). Clinical Features and Outcomes of Patients with Elizabethkingia meningoseptica Infection: An Emerging Pathogen. https://www.accjournal.org/journal/view.php?doi=10.4266/acc.2020.01158.

[19] Ahmed K., Qudsia S.A., Rehman A., Abidi S.H. (2018). Fatal Elizabethkingia meningoseptica Cholangitis Following Biliary Stent Placement. https://ecommons.aku.edu/pakistan_fhs_mc_bbs/335/.

[20] Chawla K., Gopinathan A., Varma M., Mukhopadhyay C. (2015). *Elizabethkingia meningoseptica* outbreak in intensive care unit. J. Glob. Infect. Dis..

[21] Alfouzan W., Dhar R., Al-Hashemi H., Al-Sweih N., Albert M.J. (2014). Clinical and microbiological characteristics of *Chryseobacterium* spp. isolated from neonates in Kuwait. JMM Case Rep..

[22] Chen C.-H., Lin C.-H., Lin J.-S. (2017). Bacteremia caused by *Elizabethkingia meningoseptica* in a mechanically ventilated patient successfully treated with imipenem-cilastatin and ciprofloxacin. Rev. Inst. Med. Trop. São Paulo.

[23] Chen S., Soehnlen M., Blom J., Terrapon N., Henrissat B., Walker E.D. (2019). *Comparative genomic* analyses reveal diverse virulence factors and antimicrobial resistance mechanisms in clinical *Elizabethkingia meningoseptica* strains. PLoS ONE.

[24] Meza B., Ascencio F., Sierra-Beltrán A.P., Torres J., Angulo C. (2017). A novel design of a multi-antigenic, multistage and multi-epitope vaccine against *Helicobacter pylori*: An in silico approach. Infect. Genet. Evol..

[25] Lin J.-N., Lai C.-H., Yang C.-H., Huang Y.-H. (2019). *Elizabethkingia* infections in humans: From genomics to clinics. Microorganisms.

[26] Yum J.H., Lee E.Y., Hur S.-H., Jeong S.H., Lee H., Yong D., Chong Y., Lee E.-W., Nordmann P., Lee K. (2010). Genetic diversity of chromosomal metallo-β-lactamase genes in clinical isolates of *Elizabethkingia meningoseptica* from Korea. J. Microbiol..

[27] Chen G.-X., Zhang R., Zhou H.W. (2006). Heterogeneity of metallo-β-lactamases in clinical isolates of *Chryseobacterium meningosepticum* from Hangzhou, China. J. Antimicrob. Chemother..

[28] Vessillier S., Docquier J.-D., Rival S., Frere J.-M., Galleni M., Amicosante G., Rossolini G.M., Franceschini N. (2002). Overproduction and biochemical characterization of the *Chryseobacterium meningosepticum* BlaB metallo-β-lactamase. Antimicrob. Agents Chemother..

[29] Lin X.-H., Xu Y.-H., Sun X.-H., Huang Y., Li J.-B. (2012). Genetic diversity analyses of antimicrobial resistance genes in clinical *Chryseobacterium meningosepticum* isolated from Hefei, China. Int. J. Antimicrob. Agents.

[30] González L.J., Vila A.J. (2012). Carbapenem resistance in *Elizabethkingia meningoseptica* is mediated by metallo-β-lactamase BlaB. Antimicrob. Agents Chemother..

[31] Güngör S., Özen M., Akinci A., Durmaz R. (2003). A *Chryseobacterium meningosepticum* outbreak in a neonatal ward. Infect. Control Hosp. Epidemiol..

[32] Hoque S., Graham J., Kaufmann M., Tabaqchali S. (2001). Chryseobacterium (*Flavobacterium*) *meningosepticum* outbreak associated with colonization of water taps in a neonatal intensive care unit. J. Hosp. Infect..

[33] Hsueh P.-R., Teng L.-J., Yang P.-C., Ho S.-W., Luh K.-T. (1997). Susceptibilities of *Chryseobacterium indologenes* and *Chryseobacterium meningosepticum* to cefepime and cefpirome. J. Clin. Microbiol..

[34] Vakili B., Nezafat N., Hatam G.R., Zare B., Erfani N., Ghasemi Y. (2018). Proteome-scale identification of *Leishmania infantum* for novel vaccine candidates: A hierarchical subtractive approach. Comput. Biol. Chem..

[35] Doria-Rose N.A., Joyce M.G. (2015). Strategies to guide the antibody affinity maturation process. Curr. Opin. Virol..

[36] Kumar Pandey R., Ojha R., Mishra A., Kumar Prajapati V. (2018). Designing B-and T-cell multi-epitope based subunit vaccine using immunoinformatics approach to control *Zika virus* infection. J. Cell. Biochem..

[37] Air G.M. (2015). Influenza virus antigenicity and broadly neutralizing epitopes. Curr. Opin. Virol..

[38] Solanki V., Tiwari M., Tiwari V. (2019). Prioritization of potential vaccine targets using comparative proteomics and designing of the chimeric multi-epitope vaccine against *Pseudomonas aeruginosa*. Sci. Rep..

[39] Magrane M. (2011). UniProt Knowledgebase: A hub of integrated protein data. Database.

[40] Uddin R., Siddiqui Q.N., Azam S.S., Saima B., Wadood A. (2018). Identification and characterization of potential druggable targets among hypothetical proteins of extensively drug resistant *Mycobacterium tuberculosis* (XDR KZN 605) through subtractive genomics approach. Eur. J. Pharm. Sci..

[41] Mahram A., Herbordt M.C. Fast and Accurate NCBI BLASTP: Acceleration with Multiphase FPGA-Based Prefiltering. Proceedings of the 24th ACM International Conference on Supercomputing.

[42] Li W., Godzik A. (2006). Cd-hit: A fast program for clustering and comparing large sets of protein or nucleotide sequences. Bioinformatics.

[43] Zhang R., Ou H.Y., Zhang C.T. (2004). DEG: A database of essential genes. Nucleic Acids Res..

[44] Bakheet T.M., Doig A.J. (2010). Properties and identification of antibiotic drug targets. BMC Bioinform..

[45] Shuvo M.S.R., Shakil S.K., Ahmed F. (2018). Potential Drug Target Identification of *Legionella pneumophila* by Subtractive Genome Analysis: An In Silico Approach. Bangladesh J. Microbiol..

[46] Schirmer E.C., Florens L., Guan T., Yates J.R., Gerace L. (2003). Nuclear membrane proteins with potential disease links found by subtractive proteomics. Science.

[47] Gul H., Ali S.S., Saleem S., Khan S., Khan J., Wadood A., Rehman A.U., Ullah Z., Ali S., Khan H. (2020). Subtractive proteomics and immunoinformatics approaches to explore *Bartonella bacilliformis* proteome (virulence factors) to design B and T cell multi-epitope subunit vaccine. Infect. Genet. Evol..

[48] Khan S., Ali S.S., Zaheer I., Saleem S., Ziaullah, Zaman N., Iqbal A., Suleman M., Wadood A., Rehman A.U. (2020). Proteome-wide mapping and reverse vaccinology-based B and T cell multi-epitope subunit vaccine designing for immune response reinforcement against *Porphyromonas gingivalis*. J. Biomol. Struct. Dyn..

[49] Chen L., Yang J., Yu J., Yao Z., Sun L., Shen Y., Jin Q. (2005). VFDB: A reference database for bacterial virulence factors. Nucleic Acids Res..

[50] Pandey R.K., Bhatt T.K., Prajapati V.K. (2018). Novel immunoinformatics approaches to design multi-epitope subunit vaccine for malaria by investigating *Anopheles* salivary protein. Sci. Rep..

[51] Khan S., Khan A., Rehman A.U., Ahmad I., Ullah S., Khan A.A., Ali S.S., Afridi S.G., Wei D.-Q. (2019). Immunoinformatics and structural vaccinology driven prediction of multi-epitope vaccine against *Mayaro virus* and validation through in-silico expression. Infect. Genet. Evol..

[52] Rapin N., Lund O., Bernaschi M., Castiglione F. (2010). Computational immunology meets bioinformatics: The use of prediction tools for molecular binding in the simulation of the immune system. PLoS ONE.

